# Treatment of Periprosthetic Joint Infection and Fracture-Related Infection With a Temporary Arthrodesis Made by PMMA-Coated Intramedullary Nails – Evaluation of Technique and Quality of Life in Implant-Free Interval

**DOI:** 10.3389/fsurg.2022.917696

**Published:** 2022-09-02

**Authors:** Nike Walter, Susanne Baertl, Siegmund Lang, Dominik Szymski, Johannes Weber, Volker Alt, Markus Rupp

**Affiliations:** ^1^ Department of Trauma Surgery, University Medical Center Regensburg, Regensburg, Germany; ^2^ Department of Psychosomatic Medicine, University Medical Center Regensburg, Regensburg, Germany

**Keywords:** polymethyl methacrylate (PMMA), coated implants, temporary arthrodesis, quality of life, psychological outcomes, periprosthetic joint infection, fracture-related infection

## Abstract

**Background:**

Antimicrobial coating of intramedullary nails with polymethyl methacrylate (PMMA) bone cement promises infection control and stabilization for subsequent bone healing. However, when removing the implant, bone cement can debond and remain in the medullary cavity of the long bones, representing a nidus for reinfection. This work presents a technique comprising reinforcement of PMMA-coated intramedullary nails with cerclage wire to prevent such problems in patients treated for fracture-related infection (FRI) or knee periprosthetic joint infection (PJI) with a static spacer as temporary arthrodesis allowing weight-bearing in the implant-free interval. Outcomes of this surgical treatment were evaluated in terms of (i) associated complications and (ii) patient-reported quality of life.

**Methods:**

In this retrospective case series, 20 patients with PJI (*n* = 14, 70%) and FRI (*n* = 6, 30%) treated with PMMA-coated intramedullary nails reinforced with cerclage wire between January 2021 and July 2021 were included. Quality of life during the implant-free interval was evaluated with the EQ-5D, SF-36, and an ICD-10 based psychological symptom rating and compared with previously analyzed cohorts of successfully treated PJI and FRI patients in whom eradication of infection and stable bone consolidation was achieved.

**Results:**

Complications during the implant-free interval comprised a broken nail in one case (5.0%) and a reinfection in one case (5.0%). Coating-specific side effects and cement debonding during removal did not occur. The mean physical health component score of SF-36 was 26.1 ± 7.6, and the mean mental health component score reached a value of 47.1 ± 18.6. The mean EQ-5D index value was 0.36 ± 0.32 and the mean EQ-5D visual analogue scale rating was 47.4 ± 19.4. The scores were significantly lower than those in the successfully treated FRI cohort but not in the PJI cohort. The mean ICD-10-based symptom rating scores revealed psychological symptom burden on the depression scale and enhanced levels of anxiety in comparison with healed FRI and PJI patients.

**Conclusion:**

Reinforcement of PMMA bone cement-coated implants seems to be a reasonable treatment option to create a temporary arthrodesis, preventing detachment of the bone cement when the implant was removed.

**Level of Evidence**: IV.

## Introduction

Since the introduction of antibiotic-containing polymethyl methacrylate (PMMA) bone cement to reduce the rate of periprosthetic joint infections (PJI) in arthroplasty by Buchholz and Engelbrecht in the late 1960s, the application of PMMA cement as a local antibiotic carrier for infection prophylaxis and infection therapy has become established in the fields of orthopedics and trauma surgery (1–3). In addition to commercially available antibiotic-containing PMMA chains, local application of PMMA for sheathing osteosynthesis materials has been described (4, 5). The encasement of intramedullary rods with antibiotic-containing PMMA bone cement offers several advantages. In addition to high local antibiotic concentrations, these can provide stability in unconsolidated fractures and thereby, allow fracture-associated infection to heal after surgical debridement. The stability achieved may also allow early weight-bearing of the limb. Meanwhile, PMMA cement-coated implants offer an alternative to external stabilization, which is otherwise necessary in many cases. Such large cement spacers are frequently applied in orthopedic oncology surgery for the treatment of megaprosthesis infection (6). Also, in cases of PJI and fracture-related infection (FRI) - the cement coating of intramedullary rods can serve to create a temporary knee arthrodesis, allowing full weight-bearing as a special type for a static spacer in a two-staged treatment approach (7). While mobile spacers are reported to result in a better range of motion, longtime function after reimplantation of a knee endoprosthesis, and similar infection control, static spacers may allow for pain-adapted weight-bearing without the need for additional braces or casts in the implant-free interval (8). Particularly, in complex revision cases that are not infrequently accompanied by excessive bone loss, mobile spacers are not reasonably implantable, and static spacers are a useful tool to achieve infection eradication and early mobilization of the patient. In addition, when producing custom-made static spacers, the use of PMMA-coated rods or intramedullary nails has several limitations, ranging from difficulties in fabrication to problems with implant removal during follow-up procedures. In the latter, the detachment of the PMMA bone cement from the implant and, thus, the retention of the cement in the medullary canal of long tubular bones poses a challenge. The removal of cement residues deep in the medullary can be surgically complex and time-consuming. Meanwhile, a retention of biofilm-containing infected cement residues can be considered a nidus for reinfection. Therefore, a technique has been presented including reinforcement of PMMA-coated intramedullary nails with cerclage wire to prevent such problems associated with the removal (9). Many studies have focused on comparing mobile and static spacers in terms of infection eradication and knee function after reimplantation of a revision knee prosthesis (8, 10, 11). In general, treatment success is mainly defined from a surgical perspective, and, thus, the inclusion of patient-reported outcome measures plays a major role to comprehensibly determine to what extent PJI or FRI affects the patients. Hereby, especially the quality of life has become an important outcome measure (12). However, in the area of bone and joint infection, such studies are scarce. For instance, a systematic review including 93 studies on FRI outcomes identified only three articles reporting the quality of life (13). Further, most studies assessing the quality of life have incorporated a long-term study design with a follow-up time of several years (14–18). Thus, quality of life in the implant-free interval, which can be regarded as the most critical period for patients suffering from knee PJI or FRI after articular fractures requiring joint arthroplasty, has not been investigated yet. Therefore, the purpose of this study is to first investigate complications in relation to temporary arthrodesis by cerclage wire–reinforced PMMA-coated intramedullary nails. Second, the quality of life of patients in the implant-free interval is assessed.

## Materials and Methods

### Patients

In this retrospective case series (level of evidence: IV), patients treated with PMMA-coated intramedullary nails reinforced with cerclage wire in our department between January 2021 and July 2021 were included. Informed consent was obtained from all individual participants included in the study. The study was approved by the institutional ethics committee of the University Hospital Regensburg according to the Helsinki Convention (file number 20-1681-104). Patient characteristics were retrospectively retrieved from the hospital’s electronic patient files system. Treatment indications included PJI and FRI. PJI was diagnosed according to the EBJIS consensus criteria for the diagnosis of PJI (19). FRI was defined according to the definitions of the FRI consensus group published in 2018 (20). Patient characteristics [sex, age, Charlson Comorbidity Index (CCI)], ASA score, details of orthopedic implant-associated infection such as previous revisions due to infection, reinfection, causing pathogen as well as surgery reports and implant-related adverse events until reimplantation, were assessed by reviewing electronic medical records and post-operative x-rays.

### Surgical Treatment and Preparation of Custom-Made Cerclage Wire–Reinforced PMMA-Coated Intramedullary Nails

After removal of the orthopedic implants and thorough surgical debridement and irrigation of bone and soft tissue, in all cases, intramedullary humerus nails were used from the same manufacturer (T2, Stryker, Duisburg, Germany) for arthrodesis. All implants of the T2 humeral nailing system are cannulated and made of Type II anodized titanium alloy (Ti6AL4V) (https://www.stryker.com/us/en/trauma-and-extremities/products/t2-standard-humeral-nail.html). Different other osteosynthesis materials are generally suitable for internal stabilization (21). Manufactured intramedullary humerus nails, however, have several advantages compared with simple smooth intramedullary rods. The used humeral nails have different holes and also a thread at the proximal end. The holes can additionally be used for locking of the nails if necessary. The thread at the proximal end can be used to fix an extraction instrument when nail removal with a forceps is simply not possible. This can save valuable surgical time, and to the best of our knowledge, this a major advantage that outweighs the slightly higher implant costs. All humeral nails have been used due to their availability in small diameters of 7 mm. If intramedullary diameters allow for thicker coated nails, femoral or tibial nails can also be used as an intramedullary device.

**Figure 1:**
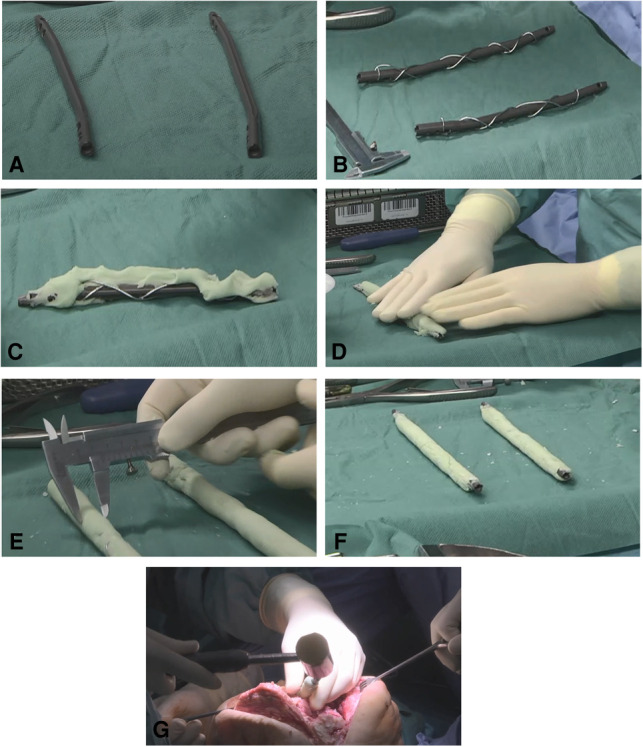
Polymethyl methacrylate (PMMA)-coated intramedullary nails. (**A, B**) 2-Humerus nails (Stryker, Duisburg, Germany) are wrapped with a 1.25- mm steel cerclage wire for reinforcement. (**C, D**) PMMA cement (Copal^®^, Heraeus Medical GmbH, Wehrheim, Germany) is applied to the nails. The hardening cement is then evenly rolled out on the instrument table. (**E, F**) The diameter is checked with the sliding gauge according to the reamed medullary canal diameter. (**G**) Reinforced PMMA-coated nails are inserted into the corresponding intramedullary canal and fixed “press fit” into the bone.

**Figure 2:**
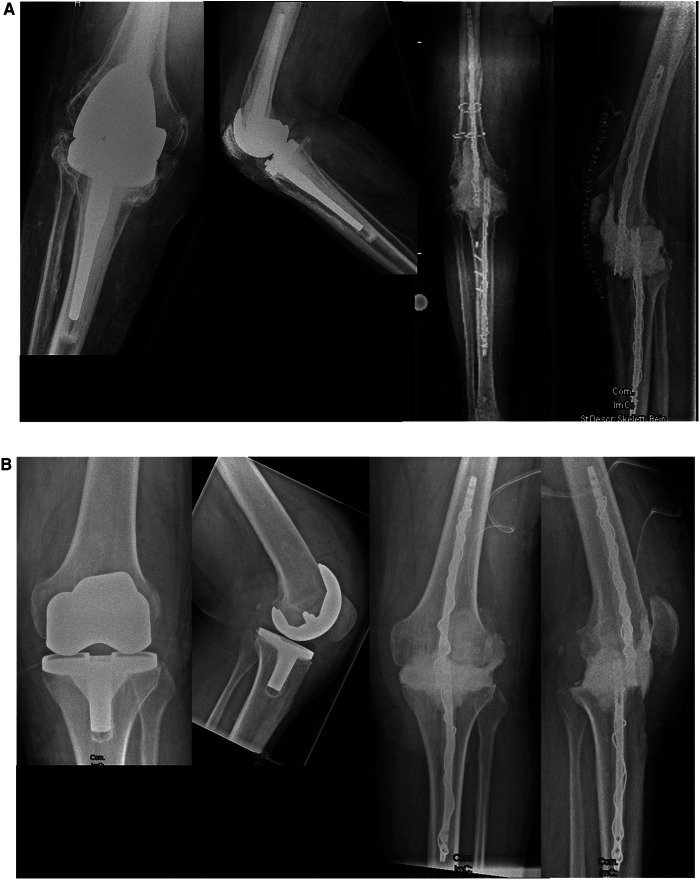
Pre-operative x-rays of (**A**) an infected rotating hinge prosthesis and (**B**) an infected bicondylar surface replacement prosthesis are shown in the left panel. Post-operative images after explantation, debridement, and temporary arthrodesis are shown in the right panel.

With the purpose of reducing the risk of cement debonding from metallic implants when removing the intramedullary implant, an additional cerclage wire was used when coating intramedullary nails (Figures 1, 2). This technique is pretty similar to the reinforcement of concrete in construction. After the bracing of a 1.25 mm cerclage wire to the intramedullary nail, PMMA bone cement can be applied to the nail. In this study, PMMA Copal^®^ (Heraeus Medical GmbH, Wehrheim, Germany) was used in all cases. After mixing the PMMA bone cement, one should wait for 3 min according to the manufacturers’ guidance before the bone cement is applied to the nail. Similar to trauma surgery, the nail diameter is adapted to the reamed intramedullary diameter. A gauge is used to measure the diameter of the coated nails. To achieve a sufficient “press fit” insertion of the coated nails, a diameter 1 mm smaller than reamed should be achieved. After hardening of the bone cement, which usually takes about 12 min, the reinforced PMMA-coated nails can be inserted into the corresponding intramedullary canal and fixed “press fit” into the bone. It is of outstanding importance to avoid implanting of still incompletely hardened cement. Immediate cement debonding can occur, and if not, bone cement can be pushed into the cancellous bone, which makes removal of the implants highly difficult in a later surgery. To achieve temporary arthrodesis of the knee, PMMA bone cement-coated nails placed in the medullary canal are overlapped in the bony defect zone of the knee. The knee is held in a flexed position of approximately 10°–15° and in a physiological leg axis. The defect zone is filled with additional PMMA bone cement. Depending on the evidenced pathogens, local antibiotics can be applied to the bone cement. When complete enclosing of the two intramedullary rods is achieved, no further connection between the two encased intramedullary nails is necessary, and patients can be allowed to bear full weight with their new temporary arthrodesis (Figure 2).

### Quality-of-Life Assessment

Patient-related outcome and quality of life was assessed using the German Short-Form 36 (SF-36) and EQ-5D scores as well as an ICD-10-based symptom rating (ISR) (22, 23). The latter is an inventory frequently used in psychosomatic anamnesis. It consists of 29 items and covers various mental syndromes with subscales for depression, anxiety, obsessive/compulsive disorders, somatoform disorders, and eating disorders (24). EQ-5D is a well-established generic quality-of-life instrument developed by the EuroQol group comprising five questions concerning the functional domains’ mobility, self-care, everyday life activities, pain/discomfort, and anxiety/depression (25). The items were converted into a single EQ index value using German norm data weights (26). Additionally, EQ-5D was evaluated using the visual analogue scale (VAS) method (27). The widely used SF-36 health survey captures the general health status with 36 questions in eight functional domains: physical function, role physical, bodily pain, general health, vitality, social function, role emotional, and mental health. Summary scores for the physical and mental component were calculated using normative data from a German national health interview and an examination survey conducted in 1998 with 7,124 participants (28). Quality of life was compared (1) with scores assessed from *n* = 37 patients after successful treatment, including eradication of infection and stable bone consolidation after long bone FRI with a follow-up of 4.2 ± 2.7 years after the last surgery (14) and (2) to scores assessed from *n* = 36 patients after successful treatment of knee PJI (mean follow-up of 4.9 ± 3.5 years (15).

Data were analyzed using SPSS statistics version 24.0 (IBM, SPSS Inc., Armonk, NY). Descriptive statistics were calculated for all variables. Continuous variables were expressed as the mean and standard deviation. For comparisons between continuous variables, independent *t*-tests were performed after determining that the distribution was appropriate for parametric testing by Levene’s test. The level of significance was set at *p* < 0.05.

## Results

In total, 20 patients (9 women, 11 men; mean age 67.3 ± 8.4 years, mean BMI 36.2 ± 9.8 kg/m^2^) were included in the analysis (Table 1). Indication for surgical treatment was PJI of the knee in *n* = 14 (70.0%) patients and FRI in *n* = 6 (30.0%) patients. The latter comprised FRI at the proximal tibia (*n* = 4) and the distal femur (*n* = 2). Two patients (10.0%) were smokers and nine patients reported to be former smokers (45.5%). The mean CCI was 3.2 ± 1.6 (range: 1–5) and the mean ASA score was 2.5 ± 0.6 (range: 1–3). The quality-of-life assessment took place after an average of 2.6 days after spacer implantation. The mean interval duration was 2.4 ± 1.6 months (range: 0.7–4.4 months).

**Table 1:** Patient characteristics.

Number	Sex	Age (years)	Indication	Pathogen
1	Male	69	PJI	*Staphylococcus hominis*, *Staphylococcus epidermidis*
2	Female	56	PJI	*S. epidermidis*
3	Male	73	PJI	*Streptococcus agalactiae*
4	Male	83	PJI	*Staphylococcus aureus*
5	Male	74	PJI	*Staphylococcus haemolyticus*, *S. epidermidis*
6	Female	71	PJI	*S. agalactiae*
7	Male	73	PJI	*Streptococcus dysgalactiae*
8	Female	72	PJI	*S. aureus*, *S. dysgalactiae*
9	Male	72	PJI	*S. aureus*
10	Female	62	PJI	*Pseudomonas aeruginosa*, *Streptococcus anginosus*, *Enterococcus faecalis*, *S. aureus*
11	Female	51	PJI	*Candida krusei*, *Serratia*, *E. faecalis*
12	Female	57	PJI	*S. haemolyticus*, *Corynebacterium amycolatum*
13	Male	68	PJI	*S. aureus*
14	Female	67	PJI	*S. aureus*, *Bacillus cereus*
15	Male	71	FRI	*S. aureus*
16	Male	61	FRI	*Enterobacter cloacae complex*
17	Female	79	FRI	*S. aureus*
18	Male	57	FRI	*S. aureus*
19	Female	73	FRI	*S. aureus*
20	Male	63	FRI	*S. epidermidis*, *Enterococcus faecium*

*PJI*, *periprosthetic joint infection*; *FRI, fracture-related infection.*

Complications during the implant-free interval occurred in two cases (10%). These comprised a broken nail in one case 21 days post-operatively (Nr. 18, Table 1). The patient had a BMI of 40.1 kg/m^2^ and was fully mobilized after the surgery. The only comorbidity of the patient was a chronic obstructive pulmonary disease. Subsequently, the spacer was exchanged. Another PJI-patient experienced a reinfection (Nr. 6, Table 1). The patient had a BMI of 51.4 kg/m^2^ and was fully mobilized with crutches after the surgery. The initial pathogen was a *Streptococcus agalactiae*, which has not been found after the implantation of the spacer. After 71 days post-operatively, *Enterococcus faecalis* and *Klebsiella pneumoniae* were identified, requiring explantation of the spacer. Coating-specific side effects and cement debonding during removal did not occur in any of the patients.

**Figure 3:**
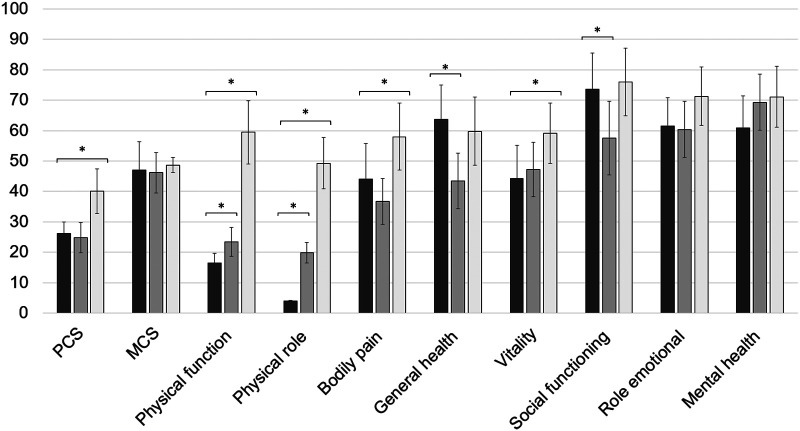
Subdimension scores for patient-related quality of life assessed with SF-36. The results of the study cohort are shown in dark gray. For a comparison, the values of the successfully treated periprosthetic joint infection (PJI) and fracture-related infection (FRI) population are illustrated in gray and light gray, respectively. * illustrates statistical significance on a *p* < 0.05 level determined by an independent *t*-test.

The mean physical health component score (PCS) of SF-36 was 26.1 ± 7.6, and its mean mental health component score (MCS) reached a value of 47.1 ± 18.6. In comparison with the successfully treated cohorts, patients with the temporary arthrodesis scored lower on the PCS than healed FRI patients did, whereas no significant difference was observed in terms of mental health. The subdomain analysis resulted in mean values of 16.6 ± 6.3 for physical function, 4.7 ± 0.8 for physical role, 44.0 ± 23.5 for bodily pain, 63.7 ± 22.4 for general health, 44.2 ± 21.9 for vitality, 73.7 ± 23.7 for social functioning, 61.4 ± 18.9 for emotional role, and 60.8 ± 21.3 for mental health (Figure 3). Here, values of the dimension physical function and physical role from the study cohort were lower than the long-term quality-of-life scores from successfully treated PJI as well as FRI patients. Interestingly, general health and social functioning were rated higher in the study cohort than in healed PJI patients, whereas bodily pain and vitality were lower than that in rehabilitated FRI patients.

**Figure 4:**
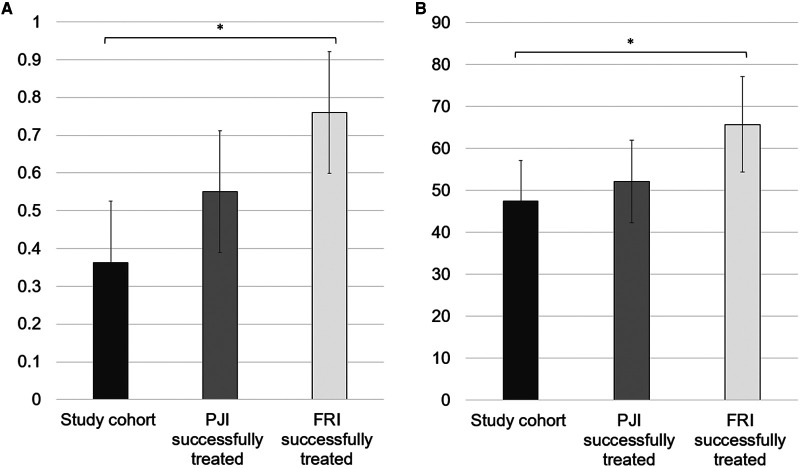
(**A**) Mean EQ-5D index value on a scale 0–1 and (**B**) mean EQ-5D VAS (visual analogue scale) rating on a scale 0–100. For a comparison, the values of the successfully treated PJI and FRI population are illustrated in gray and light gray, respectively. * illustrates statistical significance on a *p* < 0.05 level determined by an independent *t*-test.

**Figure 5:**
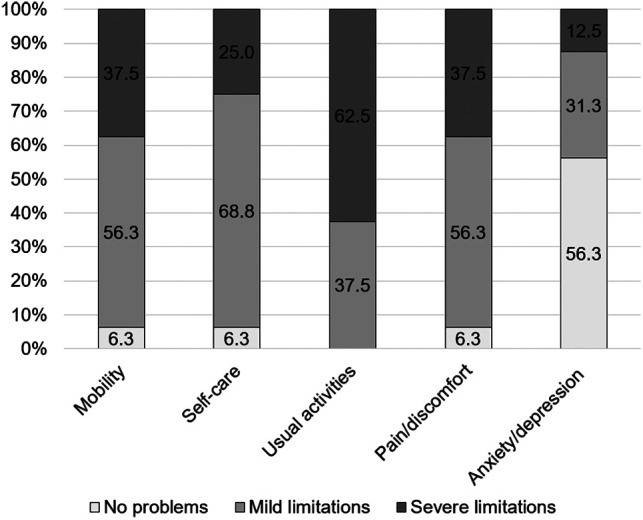
Percentage of patients showing limitations in the mobility, self-care, usual activity, pain/discomfort, and anxiety/depression of the EQ-5D subdimensions. The share of mild limitations is shown in gray, the share of severe limitations is in dark gray, and the share of no limitations is in light gray.

The mean EQ-5D index value was 0.36 ± 0.32. The mean EQ-5D VAS rating reached 47.4 ± 19.4. The EQ-5D index value, as well as the VAS rating, was significantly lower than that in the successfully treated FRI cohort (*p* < .001 and *p* = .006, respectively) (Figure 4). In the subdimensions of EQ-5D, patients showed limitations, especially concerning their everyday life activities. In total, 93.8% of the patients reported problems with mobility, self-care, and pain/discomfort. For all patients, problems with usual activities were noted, and 43.8% of patients reported limitations due to anxiety/depression (Figure 5).

**Figure 6:**
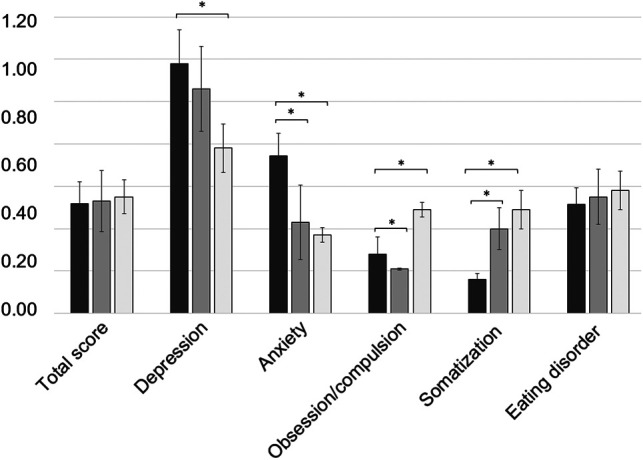
Mean values of the ISR (ICD-10-based symptom rating) total scores and depression, anxiety, obsession/compulsion, somatization, and eating disorder of the subdimensions. The values of the study cohort are shown in dark gray. For a comparison, the values of the successfully treated PJI population are illustrated in gray, and the values of the successfully treated FRI population are in light gray. * illustrates statistical significance on a *p* < 0.05 level determined by an independent *t*-test.

The mean total score of the ISR was 0.52 ± 0.20. The mean ISR subdimension scores reached 1.18 ± 0.32 for depression, 0.74 ± 0.22 for anxiety, 0.28 ± 0.16 for obsessive/compulsive disorders, 0.16 ± 0.05 for somatoform disorders, and 0.52 ± 0.15 for eating disorders, respectively (Figure 6). On average, the cohort crossed the threshold of mild symptom burden with regard to the scale depression, whereas none of the values of the other syndrome scales met the criteria for caseness, i.e., clinically relevant severity of psychological disorders. Here, depression scores were significantly higher than those in successfully treated FRI patients (*p* < 0.001), whereas patients treated with the temporary arthrodesis reached enhanced scores for the level of anxiety, as well as for the scale of obsession/compulsion, and somatization compared with healed PJI and FRI patients.

## Discussion

In this study, the technique of a temporary knee arthrodesis created with cerclage wire–reinforced PMMA-coated intramedullary nails was introduced and the quality of life during the implant-free interval was evaluated in a cohort of knee PJI and FRI patients.

### Limitations

This study shows several limitations. First, the case series includes patients with PJI and FRI as indications for surgical treatment with comparatively low case numbers. Due to the small sample size, subgroup analysis is not deemed feasible as results may be statistically underpowered. Second, the focus of the study is to evaluate patients’ quality of life during the implant-free interval, and, thus, no longer follow-ups and patient-reported outcome assessment after reimplantation or stable bone consolidation are seen. To overcome this limitation, the quality-of life scores were compared with previously analyzed successfully treated FRI and PJI patients.

Here, neither the summary scores of the SF-36 nor EQ-5D values showed a significant difference in comparison with the successfully treated PJI cohort followed up after 4.9 years on average. In line with these findings, it has been shown that patients treated with an arthrodesis showed a difference of only −1.82 points in the PCS and −3.56 points in the MCS of SF-36 in comparison with patients treated with debridement, antibiotics, and implant retention and a one-stage or two-stage exchange and, thus, knee arthrodesis might be deemed as a therapeutic alternative in cases with recurrent infections from a patient perspective (15). Here, 37.5% of the patients reported severe limitations with mobility, which seems low, considering that the scores were assessed 2.6 days on average after spacer implantation surgery and highlights the advantage of possible weight-bearing of the limb during the implant-free interval. In previous studies, functional scores and the range of motion were reported to be significantly better in patients treated with articulating spacers in comparison with patients with a static spacer, whereas the quality of life, as shown by the EQ-5D, was comparable (10).

The explicit psychological screening revealed enhanced levels of depression and anxiety in the study cohort. PJI and especially, a two-staged treatment, puts a high burden on the patients, leading to psychological distress and fears such as losing independency or experiencing a reinfection (29, 30). The need for psychological support has been explicitly reported by PJI patients. However, it should be noted that the psychological impact of PJI treatment is underestimated in the literature, and hitherto, no adequate strategies such as support interventions to address the mental burden of musculoskeletal infections have been investigated (31, 32).

The main benefit of the presented technique is that a variety of antibiotics can be added to PMMA cement according to the susceptibility of the underlying pathogen (33). The application of local antibiotic carriers in the form of implant coating is a feasible approach to bypass the unwanted side effects of systemic antibiotic therapy. Further, high local antibiotic concentrations can be reached, which is particularly required once a mature biofilm is established and persister cells are formed (34). Besides the advantages of the antibiotic coating, also in light of antibiotic stewardship, the temporary arthrodesis provides stability and allows early weight-bearing of the limb. Thus, especially in cases of unconsolidated fractures, the procedure provides an alternative to external stabilization.

For cement-coated intramedullary implants, removal can be highly challenging in follow-up surgeries. Debonded cement residues deep in the medullary can be surgically difficult to remove. This, in general, is time-consuming and the bone is at risk of experiencing an iatrogenic fracture. The complication of cement debonding was reported in 23 out of 110 cases (21%) with infected nonunions treated with antibiotic cement-coated rods (35). Also, other authors reported problems with removal in 10%–25% of patients (5, 36–39). It has been suggested to remove cement debonds with a J-hook or with sequential reaming and subsequent copious irrigation of the canal using canal tip pulsed lavage in case removal fails. Often, a distal vent channel or bone fenestration is required to completely remove retaining cement (36, 40). Thus, the presented technique of reinforcement of PMMA-coated intramedullary nails with cerclage wire is beneficial for preventing such problems. Also, other techniques for preventing cement debonding have been reported, such as using threaded cores or roughening the nail surface before coating to enhance the adherence of cement (41). To note, no specific guidelines exist with regard to the techniques used to cement-coat implants in a custom-made fashion, and there is a considerable heterogeneity in the reported literature, making it challenging to arrive at a general consensus (21). In the same way, the superiority of reinforced versus unreinforced implants with regard to post-operative complications has yet be proven due to the lack of randomized comparative studies. A broken nail occurred in one case (5.0%), which has also been reported by other authors (5, 21, 37, 42). For instance, Qiang et al. used a self-made antibiotic cement rod for the treatment of intrameduallary infection reporting one broken rod (5.3%) and one complication during removal due to a too large diameter of the rod (5.3%) (36). Paley and Herzenberg performed a preliminary study with *n* = 9 cases treated with a custom-made antibiotic-impregnated cement rod for diverse indications. In one patient with an infected nonunion of the humerus, the rod broke after 2 years (5). In another cohort consisting of 67 patients with an infected arthrodesis, a broken rode (diameter 10 mm) occurred in one case (1.5%) (35).

Here, no coating-specific side effects and cement debonding during removal were observed. Notably, orthopedic surgeons have been hesitant to combine stainless steel and titanium implants due to concerns of galvanic corrosion (43). However, multiple studies have shown no clinical complications or a negative influence when mixing stainless steel and titanium implants for osteosynthesis (44–47). Thus, the reinforcement of bone cement-coated implants seems to be a beneficial option.

## Conclusion

Reinforcement of PMMA bone cement-coated implants seems to be a reasonable treatment option to create a temporary arthrodesis to prevent detachment of the bone cement when the implant is removed.

## Data Availability

The raw data supporting the conclusions of this article will be made available by the authors, without undue reservation.
